# Potential to Ensure Safe Production of Water Spinach in Heavy Metals-Contaminated Soil by Substituting Chemical Fertilizer with Organic Fertilizer

**DOI:** 10.3390/plants13202935

**Published:** 2024-10-20

**Authors:** Hao Qi, Zhong Zhuang, Jiang Liu, Siyu Huang, Qiqi Wang, Qi Wang, Huafen Li, Yanan Wan

**Affiliations:** Beijing Key Laboratory of Farmland Soil Pollution Prevention and Remediation, College of Resources and Environmental Sciences, China Agricultural University, Beijing 100193, China; qihao_blake@163.com (H.Q.); zhuangzhong@cau.edu.cn (Z.Z.); 15548810038@163.com (J.L.); huangsiyu@cau.edu.cn (S.H.); wangqiqi7777888@163.com (Q.W.); wangqi88@cau.edu.cn (Q.W.); lihuafen@cau.edu.cn (H.L.)

**Keywords:** organic fertilizer, heavy metals, water spinach, safe production

## Abstract

Organic fertilizers are widely used to improve soil quality. However, their potential for ensuring the safe production of vegetables in soils with varying levels of heavy metals pollution remains inadequately explored. Here, we conducted a pot experiment to investigate the effects of substituting chemical fertilizers with organic fertilizer on the HMs accumulation in water spinach by simulating soils with different levels of HMs pollution. The results showed that the organic fertilizer significantly increased the soil pH, cation exchange capacity (CEC), and organic matter (OM). Furthermore, it led to a reduction in the soil DTPA–Cd and DTPA–Pb levels by 3.3–20.6% and 22.4–47.3%, respectively, whereas the DTPA–As levels increased by 0.07–7.7 times. The organic fertilizer effectively reduced the Cd and Pb content in water spinach below the safety limits when the added Cd content in the soil was less than 2 mg/kg and the Pb content was equal to or less than 90 mg/kg. However, its efficacy in reducing As accumulation in water spinach was limited, emphasizing the need for caution when using organic fertilizers in As-contaminated soils. Our results provide valuable insights for the scientific and precise utilization of organic fertilizers, thereby contributing to the safe production of vegetables.

## 1. Introduction

Soil is a crucial sink for HMs, and maintaining clean agricultural soil is imperative for ensuring food safety [1]. However, the intensive use of chemical fertilizers in agricultural production has led to soil acidification [2], potentially remobilizing the original HMs in the soil and endangering agricultural products’ safety. Consequently, effective strategies to reduce chemical fertilizer usage and mitigate soil contamination are urgently needed. Vegetables are crucial components of human diets [3]. Prolonged consumption of HMs-contaminated food may lead to their accumulation in human organs, causing toxicity and serious health issues [4,5,6,7]. Notably, leafy vegetables exhibit stronger ability for HMs accumulation than other vegetable types [8,9]. Therefore, ensuring the safe production of leafy vegetables becomes paramount for human health.

Various techniques have been used to remediate soil contaminated with HMs. Soil turnover and phytoremediation aim to remove HMs from the soil, but they are costly or take a long time, making it difficult to widely adopt them in HMs-contaminated farmland [10]. By contrast, amendments are widely used in HMs-contaminated farmland to immobilize the HMs, such as lime, clay minerals, phosphate minerals, biochar, and organic fertilizer [11,12,13,14,15]. Among these amendments, organic fertilizers stand out as a cost-effective option with a minimal impact on the soil structure, promoting vegetable production [16,17,18]. Therefore, organic fertilizers emerge as a preferred option for the remediation of HMs-contaminated farmland.

Organic fertilizers can introduce a substantial presence of humic substances [19]. The carboxyl and phenolic groups of humic substances can form coordination complexes with metal ions, exerting a crucial influence on metal complexation in soil [20,21,22]. Moreover, it is well known that the application of organic fertilizers can have a significant impact on the soil pH [11,23]. The soil pH is a critical variable influencing the adsorption of metal cations by oxidized minerals, directly and indirectly impacting the availability of HMs in soil [24,25,26]. However, most studies only focus on the remediation effect of organic fertilizers on soils contaminated with only one specific content of heavy metals [27,28,29], while the remediation effects of organic fertilizers may vary in soils with different contents of heavy metals pollution. It seems that decision-makers and farmers are more concerned about what measures to take at different levels of pollution. Therefore, we aim to explore the critical contents of HMs in soil that enable the safe production of water spinach by using organic fertilizers.

Water spinach (*Ipomoea aquatica* Forsk.), a widely grown vegetable in China, was chosen as the model crop for this study due to its short growing cycle and susceptibility to HMs contamination [30]. We conducted a pot experiment using six artificially prepared composite-polluted soils to simulate various HMs-contaminated scenarios, aiming to (1) investigate the growth and HMs accumulation characteristics (including cadmium (Cd), arsenic (As), and lead (Pb)) of water spinach under different fertilization regimes; (2) analyze the impact of organic fertilizer application on the soil properties and HMs availability; and (3) elucidate the interaction between soil properties, HMs, and HMs accumulation in water spinach.

## 2. Materials and Methods

### 2.1. Preparation of HMs Composite Contaminated Soil

The tested soil was collected from the surface layer (0–20 cm) in Daolang town, Tai’an City, Shandong Province, China. The soil was air-dried and passed through a 2 mm sieve to ensure a uniform particle size. It exhibited the following properties: pH 6.1, cation exchange capacity (CEC) 10.92 cmol/kg, organic matter (OM) 24.19 g/kg, total nitrogen 0.64 g/kg, available phosphorus 48.1 mg/kg, available potassium 408.4 mg/kg, total Cd 0.16 mg/kg, total As 10.43 mg/kg, total Pb 21.09 mg/kg. Six contamination levels of heavy metals (HMs) were established (Table 1), considering the risk screening value (RSV) and risk intervention value (RIV) for soil with a pH between 5.5 and 6.5, as specified in the GB 15618-2018 [31].

To incorporate the HMs into the soil, a solution containing the HMs was thoroughly mixed with the soil manually to ensure a homogeneous distribution. The reagent was prepared as a mother liquor with a consistent content, and deionized water was added to ensure that the volume of the added liquid was the same for all the treatments. The amounts of Cd added (calculated as Cd) were 0, 0.1, 0.3, 1.0, 2.0, and 5.0 mg/kg, while the amounts of As added (calculated as As) were 0, 20, 40, 100, 150, and 200 mg/kg. Similarly, the amounts of Pb added (calculated as Pb) were 0, 50, 90, 300, 500, and 1000 mg/kg. The Cd, As, and Pb were added to the soil in the form of Cd(NO_3_)_2_·4H_2_O, Na_2_HAsO_4_·7H_2_O, and Pb(NO_3_)_2_, respectively. After the addition of the HMs, the soil was thoroughly mixed and aged in pots for 2.5 years. Each contamination level was replicated three times to ensure the reliability of the results.

### 2.2. Pot Experiments

The tested chemical fertilizers were urea (N 46.6%), potassium chloride (K_2_O 62.9%), and calcium superphosphate (P_2_O_5_ 19.2%). The basic properties of the tested organic fertilizer (pig manure compost) were as follows: pH 8.4, organic matter 768 g/kg, total nitrogen 24.2 g/kg, total phosphorus 15.9 g/kg, total potassium 15.5 g/kg, total Cd 0.51 mg/kg, total As 11.0 mg/kg, total Pb 8.70 mg/kg. The experiment employed three fertilizer treatments, and the specific fertilizer application amounts are shown in Table 2. The fertilizers were meticulously mixed with the soil to ensure a uniform distribution. Subsequently, the flowerpots were kept at approximately 75% field moisture capacity for one week to achieve a soil moisture balance. Each pot contained 3.0 kg of soil, and each treatment had three replicates.

Water spinach seeds were disinfected with 10% H_2_O_2_ for 30 min to eliminate any potential contaminants and soaked in saturated calcium sulfate for 4 h to enhance their germination. Finally, the seeds were washed with deionized water to ensure cleanliness and sown into pots after 12 h of soaking in deionized water. After about a week, when the seedlings of water spinach grew to the appropriate size, the seedlings were carefully thinned out to six plants per pot. During the experimental period, deionized water was added to compensate for evaporation and transpiration. The growth conditions of water spinach were as follows: temperature at 25 ± 5 °C/20 ± 2 °C (day/night) under a 14 h photoperiod with a light intensity of 240–350 µmol/m^2^/s and relative humidity of 60–70%. After 40 d of incubation, samples of the edible parts of water spinach and soil were collected simultaneously for further analysis.

### 2.3. Plant Sampling

The water spinach shoots were first separated, weighed, and then frozen in liquid nitrogen. They were subsequently pulverized and stored at −20 °C. Fresh shoot samples weighing 0.4000 g were digested with 8 mL of concentrated HNO_3_ (GR) using an electrothermal digester (DigiBlock ED54, LabTech, Beijing, China). The digested samples were then diluted to a final volume of 50 mL with high-purity water and filtered. The concentrations of Cd, Pb, and As in the digestion solutions were analyzed using inductively coupled plasma mass spectrometry (ICP-MS 7700, Agilent Technologies, Santa Clara, CA, USA). To ensure the accuracy of the elemental analysis, blanks and certified reference material (GBW10049, GSB-27, scallion, Institute of Geophysical and Geochemical Exploration, Chinese Academy of Geological Sciences, Langfang, China) were utilized. The recoveries of Cd, As, and Pb ranged from 100% to 107%, 97% to 125%, and 86% to 119%, respectively.

### 2.4. Soil Sampling

After harvesting, the soil samples were air-dried at room temperature and analyzed for the pH, CEC, and available elements through a 2 mm nylon sieve, and for the soil OM through a 0.149 mm nylon sieve. The soil pH was measured using a pH meter (HI 98127, Hanna Instruments, Singapore) with a soil to deionized water ratio of 1:2.5 (*w*/*v*). The available HMs were determined by the Soil–Determination of bioavailable form of eight elements—Extraction with buffered DTPA solution/Inductively coupled plasma optical emission spectrometry [32]. The soil CEC was determined according to the Soil Quality—Determination of cation exchange capacity (CEC)—Hexamminecobalt trichloride solution—Spectrophotometric [33]. The soil organic matter (OM) was determined by the method of potassium dichromate external heating.

### 2.5. Data Analysis

The experimental data were processed using Microsoft Excel 2016, and the processed data and correlation analysis were visualized using Origin 2024 software. The mean values ± standard errors (n = 3) are presented, and the statistical analysis was performed using SPSS 25.0 software. A one-way analysis of variance (ANOVA) followed by Duncan’s multiple range test (*p* < 0.05) was conducted to assess the significance between the different treatments. Heatmap visualization was generated using the “pheatmap” package in R 4.3.2. Additionally, conditional inference trees (CITs) analysis was carried out using the “party” package in R 4.3.2 to identify key factors and conditions influencing the accumulation of heavy metals in water spinach.

The bioconcentration factor (BCF) of the heavy metals in water spinach was calculated using Equation (1):

BCF = *C*_water spinach_/*C*_soil_ (1)

where *C*_water spinach_ is the HMs content in the edible parts of water spinach, which is calculated by the fresh weight; and *C*_soil_ is the total HMs content in the soil, which is the sum of the contents of exogenous added HMs and the soil background value.

## 3. Results

### 3.1. Biomass of Edible Parts of Water Spinach

**Figure 1:**
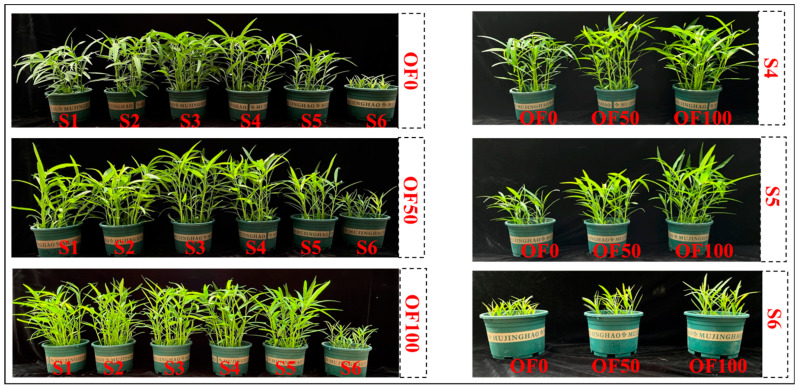
Growth of water spinach before harvest.

The biomass results indicated a significant inhibitory effect on the growth of water spinach due to the stress induced by high contents of HMs (Table 3). In the OF0 treatment, significant toxic effects, characterized by leaf chlorosis and slowed or halted growth, were observed in water spinach in S5 and S6 soil (Figure 1). In the OF0 treatment, the biomass of water spinach in S5 and S6 soils decreased by 51.6% and 90.8%, respectively, compared to that in S1 soil. However, the application of organic fertilizer alleviated the stress of high contents of HMs on the water spinach, with the mitigation effect becoming more pronounced with increasing HMs levels in the soil. Specifically, the OF50 and OF100 treatments increased the biomass of water spinach in S4 soil by 30.0% and 43.8%, respectively. Similarly, in S5 soil, the biomass of water spinach increased by 90.7% and 155%, respectively, compared to the OF0 treatment. Furthermore, it was observed that low-dose HMs promoted the growth of water spinach, while high-dose HMs inhibited it. In the OF0 treatment, water spinach exhibited the highest biomass in S3 soil, with a significant difference compared to that in S1 and S6 soils (*p* < 0.05).

### 3.2. HMs Accumulation in Water Spinach

**Figure 2:**
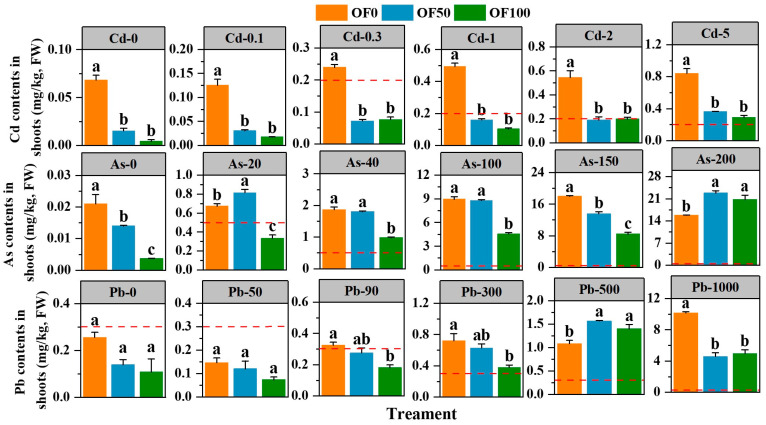
Cd, As, and Pb contents in the edible parts of water spinach. The red dotted lines represent the limits of the national food safety standard (GB 2762-2022). Different lowercase letters represent significant differences for different fertilizer treatments (*p* < 0.05).

Within the experimental dose range, the Cd, As, and Pb content in the edible parts of the water spinach increased with the increase of Cd, As, and Pb in the soil, ranging from 0.004 to 0.84, 0.004 to 22.83, and 0.074 to 10.17 mg/kg, respectively (Figure 2). In the OF0 treatment, when the Cd, As, and Pb content added to the soil reached 0.3, 20, and 90 mg/kg, respectively, the Cd, As, and Pb levels in the edible parts of the water spinach exceeded the limits of the national food safety standard in GB 2762-2022 [34].

The application of organic fertilizer demonstrated varying effects on the accumulation of Cd, As, and Pb in water spinach. Organic fertilizer had an excellent effect on reducing Cd in the edible parts of water spinach. Specifically, compared to the OF0 treatment, both the OF50 and OF100 treatments significantly reduced the Cd content in the edible parts of water spinach in all the polluted soils (*p* < 0.05), with the reductions ranging from 56.7% to 77.5% and 63.6% to 93.5%, respectively. Moreover, both OF50 and OF100 treatments could reduce the Cd content in the edible parts of water spinach below the limits of the national food safety standard when the Cd content added in soil was below the risk intervention value of 2 mg/kg. Regarding As, under four As treatments (0, 40, 100, and 150 mg/kg), the OF50 and OF100 treatments reduced the As content in the edible parts of the water spinach by 1.7–33.3% and 47.6–82.1%, respectively. Under four Pb treatments (0, 50, 90, and 300 mg/kg), compared with the OF0 treatment, both the OF50 and OF100 treatments reduced the Pb content in the water spinach, with a reduction range of 13.0–45.3% and 47.3–57.6%, respectively.

**Figure 3:**
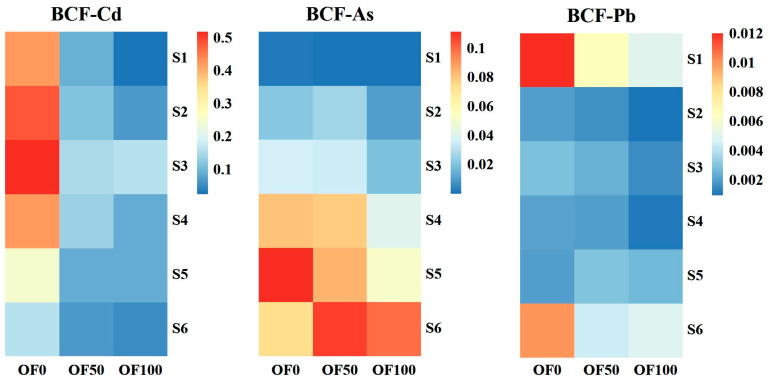
The bioconcentration factor (BCF) for the Cd, As and Pb content of water spinach under different treatments.

Moreover, the bioconcentration factor (BCF) results demonstrated significant effects of different fertilization regimes on the accumulation of HMs in water spinach (Figure 3). Furthermore, distinct accumulation characteristics were observed for different HMs in water spinach, with the plant exhibiting a higher tendency to accumulate Cd compared to As and Pb. Specifically, the BCF values for Cd, As, and Pb in water spinach ranged from 0.03 to 0.52, 0.0004 to 0.11, and 0.0012 to 0.012, respectively.

### 3.3. Effect of Organic Fertilizer on Soil Properties and the Available HMs

**Figure 4:**
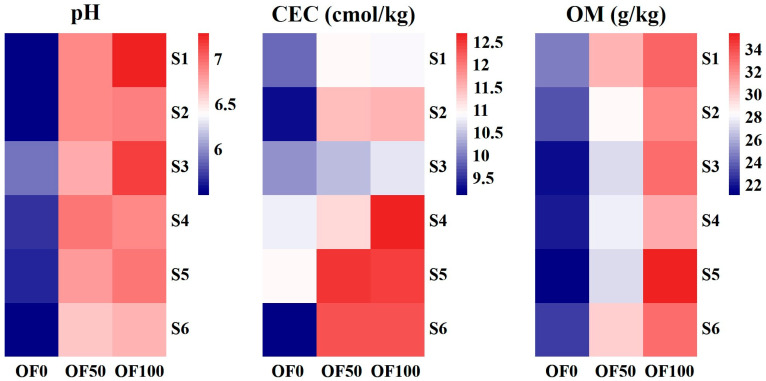
The soil pH, CEC, and OM under different treatments.

The soil pH, cation exchange capacity (CEC), and organic matter (OM) under different treatments are shown in Figure 4. The application of organic fertilizer caused a significant increase in the soil pH, CEC and OM. The OF50 and OF100 treatments led to an average increase of 1.17 and 1.34 units in the soil pH, respectively, along with average increases of 12.6% and 17.3% in the soil CEC, and 25.5% and 45.1% in the soil OM, respectively.

**Figure 5:**
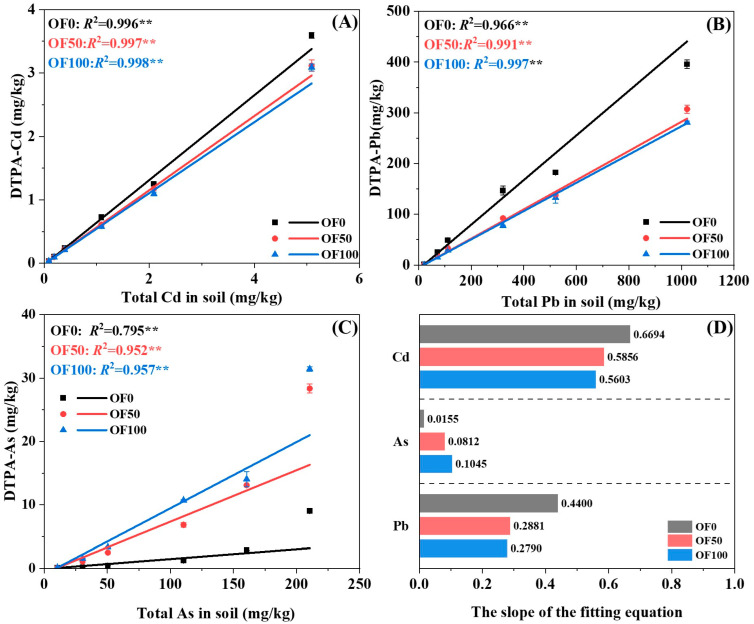
Linear fitting of the total HMs and DTPA–HMs in soil (**A**–**C**) and the fitting equation slopes (**D**). ** indicates a significance level of *p* < 0.01.

The content of available HMs in the soil increased as the content of HMs added to the soil increased (Figure 5). Specifically, the content of available Cd in the soil ranged from 0.03 to 3.60 mg/kg, 0.03 to 3.11 mg/kg, and 0.03 to 3.09 mg/kg in the OF0, OF50, and OF100 treatments, respectively. Both the OF50 and OF100 treatments resulted in decreased available Cd content compared to the OF0 treatment, with the reductions ranging from 3.3% to 16.2% and 5.5% to 20.6%, respectively. The available As content ranged from 0.08 to 9.03 mg/kg, 0.08 to 28.4 mg/kg, and 0.11 to 31.4 mg/kg in the OF0, OF50, and OF100 treatments, respectively. The OF50 and OF100 treatments significantly increased the available As content compared to OF0 treatment, with increases of 0.07 to 5.0 times and 0.49 to 7.7 times, respectively. The available Pb content ranged from 0.81 to 396 mg/kg, 0.58 to 307 mg/kg, and 0.59 to 281 mg/kg in the OF0, OF50, and OF100 treatments, respectively. Similar to the results observed for Cd, both the OF50 and OF100 treatments led to significant reductions in the available Pb content compared to the OF0 treatment, with the reductions ranging from 22.4% to 37.1% and 27.0% to 47.3%, respectively.

Additionally, the results of the linear fitting also revealed a significant positive correlation between the available and total contents of Cd, As, and Pb in the soil (*p* < 0.01) (Figure 5A–C). The slope of the fitting equation can be used to assess the bioavailability of HMs in soil under different fertilization regimes (Figure 5D). For Cd and Pb, the slope of the fitting equation was found to be OF0 > OF50 > OF100. Contrarily, the slope of the fitting equation between the available and total As content showed OF100 > OF50 > OF0. Thus, the application of organic fertilizer was found to reduce the bioavailability of Cd and Pb in soil, while increasing the bioavailability of As.

### 3.4. The Interactive Effects of HMs and Soil Properties on the HMs Accumulation in Water Spinach

**Figure 6:**
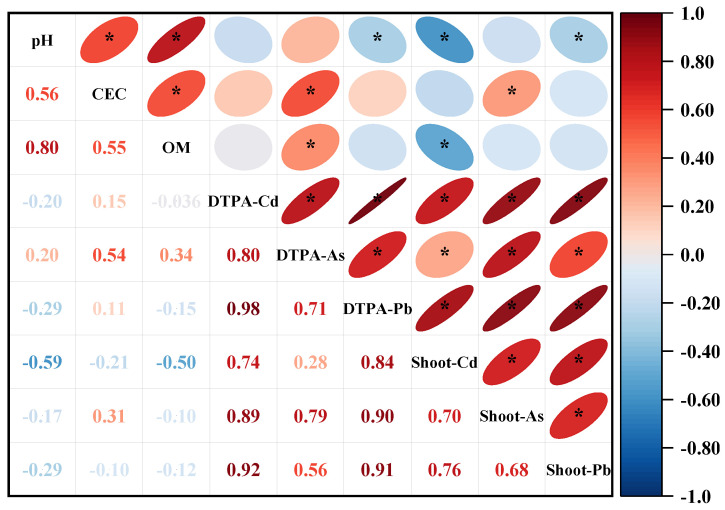
Pearson correlations between the soil properties, soil available HMs content and shoot HMs content of water spinach. * indicates a significance level of *p* < 0.05, n = 54.

Pearson correlations were performed to identify the critical factors in determining the shoot HMs content in water spinach (Figure 6). The results showed that the shoot Cd content of water spinach was significantly negatively correlated with the soil pH (*r* = −0.59, *p* < 0.05) and soil OM (*r* = −0.50, *p* < 0.05). The shoot Pb content of water spinach was significantly negatively correlated with the soil pH (*r* = −0.29, *p* < 0.05), while the shoot As content of water spinach was significantly positively correlated with the soil CEC (*r* = 0.31 *p* < 0.05). In addition, the shoot Cd, As, and Pb contents of water spinach showed significant positive correlations with the contents of available Cd, As, and Pb in the soil, with correlation coefficients of 0.74, 0.79, and 0.91, respectively (*p* < 0.05).

**Figure 7:**
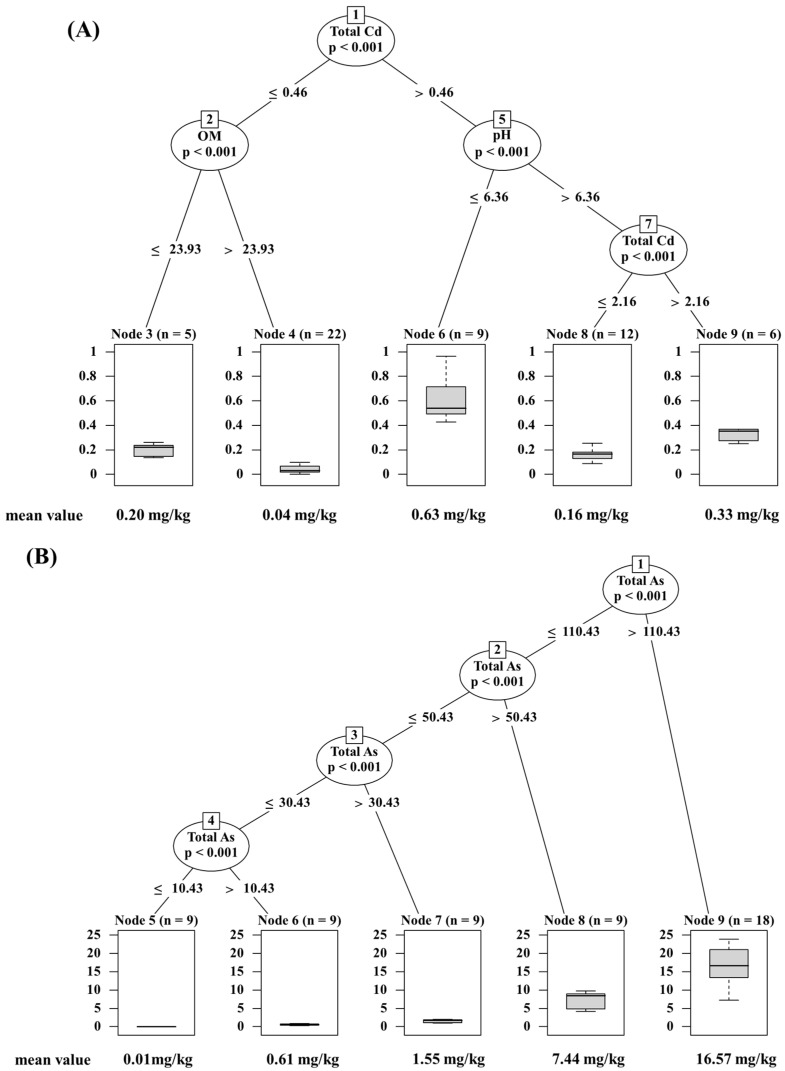

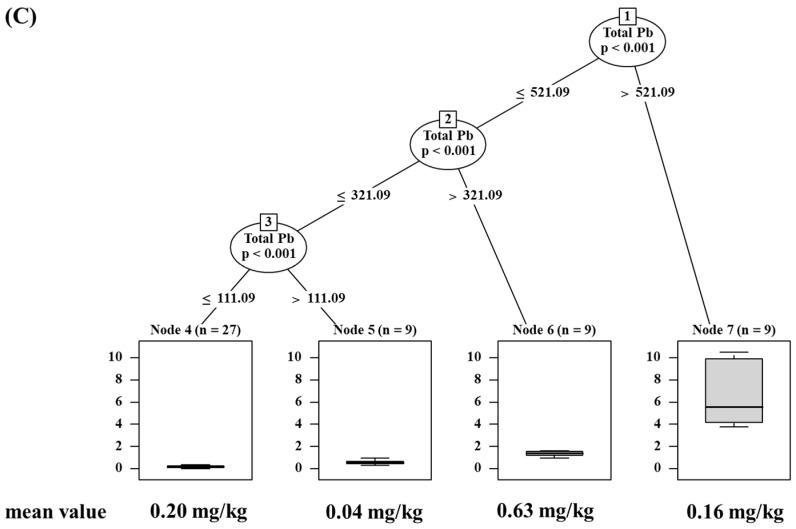
Conditional interference trees of Cd (**A**), As (**B**), and Pb (**C**) accumulation in water spinach. The values shown on the edges of the path represent the corresponding criteria. The unit for the soil HMs content is mg/kg, and that for the OM is g/kg. Box plots depict the distribution of the HMs contents in water spinach under different categories.

Conditional inference trees (CITs) were employed to ascertain the key factors and conditions influencing the accumulation of Cd, As, and Pb in water spinach (Figure 7). All the significant mappings were included in the resulting trees (*p* < 0.01). For Cd, the CITs results were categorized into five groups, with the soil total Cd, OM, and pH serving as classification factors (Figure 7A). The soil total Cd emerged as the primary splitting factor for the root nodes in the regression tree, indicating its significance in contributing to the accumulation in water spinach. The critical values for the soil total Cd were determined to be 0.46 and 2.16 mg/kg, with the soil OM at 23.93 g/kg and soil pH at 6.36, as revealed by significance testing of the CITs models. The accumulation of Cd in water spinach was notably affected by variations in the soil properties resulting from organic fertilizer application. For instance, when the soil total Cd was ≤0.46 mg/kg, the Cd content in water spinach at a soil OM ≤ 23.93 g/kg was 5.0 times higher compared to that at a soil OM > 23.93 g/kg. Similarly, when the soil total Cd was >0.46 mg/kg, the Cd content in water spinach was 3.9 times higher when the soil pH was ≤6.36 compared to when the soil pH was >6.36, and the soil total Cd was ≤2.16 mg/kg. Conversely, no significant effect of the soil properties on As and Pb accumulation in water spinach was observed, with only the soil total As and Pb identified as the primary factor (Figure 7B,C).

## 4. Discussion

### 4.1. Effect of Organic Fertilizer on Available HMs

Previous studies have extensively documented the significant impact of long-term organic fertilizer application on soil properties [23,35,36]. Numerous adsorption kinetics and adsorption isotherm experiments have confirmed the adsorption properties of organic fertilizers on HMs [37,38]. However, isolating the direct effect of organic fertilizer itself on the HMs in soil presents challenges due to its decomposition after application. Consequently, we hypothesize that organic fertilizer influences the active fraction of HMs by modifying soil properties. In this study, the application of organic fertilizer led to a notable increase in the soil pH, CEC, and OM (Figure 4).

Consistent with previous studies [23,39], the application of organic fertilizer increased the soil pH and reduced the potential bioavailability of soil Cd and Pb, instead increasing the potential bioavailability of As (Figure 5). The soil pH increased significantly with the escalating application of organic fertilizer, given the slightly acidic nature of the soil in this study and the alkaline characteristic of organic fertilizer. Compared with the background pH value of the tested soil, the treatment of applying chemical fertilizers alone showed significant acidification, which further led to a decrease in the soil acid–base buffering performance. Xiao et al. (2021) also reported that continuous application of chemical fertilizers significantly reduced the soil pH compared to non-chemical fertilization treatment, while applying manure alone or in combination with chemical fertilizers increased the soil pH [40].

In general, as the soil pH rises, the mobility and solubility of cationic heavy metals tend to decrease, while the pattern for anionic heavy metals behaves conversely [17]. Many studies have reported a negative correlation between the soil pH and the solubility of Cd and Pb in soil [41,42,43]. The decrease in the soil pH can disrupt the precipitation and dissolution balance of metal ions in the soil, promoting the release of metals from the soil solid phase to the soil solution [44]. In addition, the increase in the soil pH will increase the negative charge on the surface of soil colloids, thereby promoting the formation of iron/manganese oxides, providing more binding sites for cationic metals such as Cd and Pb [45]. However, for oxyanions such as As, the solubility might increase with an increase in the pH. The positive charges on the soil surfaces with an increasing pH lead to the desorption of As [24,46].

Soil organic matter is acknowledged as the primary factor controlling the bioavailability and chemical behavior of heavy metals (HMs) in the environment [47,48]. The application of organic fertilizer also directly introduces organic matter such as humic and fulvic acids containing a large number of functional groups into the soil [22,49,50]. The organic component of soil has a high affinity for heavy metals cations such as Cu, Cd, and Pb because of the presence of ligands or groups that can form chelates with HMs [49]. Chen et al. (2020) revealed the involvement of phenolic- and carboxylic-like groups in soil organic matter in the complexation of Pb and Cd, resulting in the formation of highly conjugated macromolecular substances with a positive impact on the reduced activity of Cd and Pb [21]. On the contrary, the application of organic fertilizer enhances the mobility and bioavailability of As. This may attributed to the elevated levels of dissolved organic carbon (DOC), which can be adsorbed onto Fe(III) (hydro)oxides (FeOOH) via ligand exchange and compete with As for active adsorption sites [51]. Furthermore, the DOC stimulates the activity of Fe(III)-reducing bacteria, promoting the microbial reduction of FeOOH and resulting in the release of As(III) into the soil solution [52].

The application of organic fertilizers can also supplement a large amount of base cations in the soil and increase the soil CEC (Figure 4), thereby improving the acid–base buffering performance and increasing metal adsorption [53]. Because the cation exchange capacity (CEC) determines soil ion-binding capacity, CEC is also considered an important factor affecting HMs mobility in soil. Soils with higher CEC generally cause increased cationic HMs binding capacity, leading to reduced HMs mobility in soil [54]. Nevertheless, distinguishing the contribution of CEC alone is challenging due to the simultaneous stimulation of soil CEC by increases in the soil pH and organic matter [11].

### 4.2. Effect of Organic Fertilizer on HMs Accumulation in Water Spinach

Plants have different defense mechanisms against selected heavy metals. The impact of organic fertilizer on the HMs accumulation in plants grown in composite-polluted soil is a complex and challenging issue, and it is hard to rule out that there may be interactions between these three HM elements in soil and plants. We found that the application of organic fertilizer had a positive effect on the growth of water spinach and that it mitigated the toxic effects at high HMs contents (Table 3 and Figure 1). In summary, organic fertilizer can effectively reduce the Cd content in water spinach in all polluted soils. When the added Cd in the soil was below the risk intervention value of 2 mg/kg, and the Pb was below or equal to the risk screening value of 90 mg/kg, the application of organic fertilizer reduced the Cd and Pb content in water spinach to below the safety limit in GB 2762-2022 (Figure 2). However, when the As added to the soil has not yet reached the risk screening value of 40 mg/kg, the As content in water spinach exceeded the limit, and the application of organic fertilizer did not reduce it to a safe range. This may be due to the different mobility of the three elements in the soil or in the plant. As a previous study by Yu and Wu. (1997) demonstrated, Cd adsorbed by soil is mostly physisorbed, while Pb is mostly chemisorbed, about 70% of Cd adsorbed by soil can be desorbed, about 50% of As can be desorbed, and Pb has the weakest mobility, as only less than 20% can be desorbed [55]. Moreover, the results of the bioconcentration factor (BCF) also indicated that water spinach exhibited a stronger bio-accumulative potential for Cd compared to As and Pb (Figure 3).

CITs were employed to further analyze the sensitivity of heavy metals (HMs) accumulation in water spinach with changes in the soil total HMs and properties. Similar to the results of the study on Cd accumulation in wheat and maize by Zhuang et al. (2021) [56], the results of the CITs in this study indicated that the total HMs in soil was the most important factor influencing the accumulation of HMs in water spinach. This suggests that the regulation of input and accumulation of toxic heavy metals in agricultural soils is of paramount importance. In addition, the Cd contents in water spinach were closely related to the interactions between the soil pH and the OM. The Cd content in water spinach was lower under high soil OM and high soil pH, which was consistent with the results of the correlation analysis (Figure 6). Thus, the accumulation of Cd by water spinach was strongly influenced by changes in the soil properties due to organic fertilizer application.

Contrary to Cd, the accumulation of As and Pb in water spinach remained unaffected by the soil properties. In this study, the variations in the soil As availability and its uptake by water spinach exhibited inconsistency. While the organic fertilizer addition increased the DTPA–As content in soil (Figure 3), it did not consistently elevate the As contents in all the treatments of water spinach (Figure 2). Similarly, Madeira et al. (2012) also found that urban garbage composting treatment can promote the growth of tomatoes on HMs-contaminated soil, improve the availability of As in contaminated soil (CaCl_2_ extraction), and reduce the As content in tomato fruits [57]. The results of Hattab et al. (2015) also indicated that the addition of sludge compost increased the mobility of As in soil (DGT extraction), but the As content in various parts of the rice significantly decreased [58]. This may be due to the dual effect of phosphate release from organic fertilizers on the As uptake by plants. In this study, the total phosphorus carried to the soil by the organic fertilizer was much higher than that of chemical fertilizers, and the phosphorus in the organic fertilizer was released slowly during the incubation process, allowing for a continuous supply of phosphorus during the growing period of the water spinach. On the one hand, phosphate and arsenate have the same adsorption sites in the soil solid phase, and phosphate can replace arsenate adsorbed in the soil solid phase, leading to an increase in the available arsenic content in the soil [59,60]. On the other hand, phosphate and arsenate also share the same transport pathway in plants. If the demand of plants for phosphorus is met, the transport protein will be downregulated, thereby inhibiting As absorption [61]. The effect of organic fertilizer on the increase in the available As content in soil may be offset by the competition between phosphorus and arsenic during plant root absorption [62,63]. This also indicated that the potential available states of HMs using extractants more reflects their active part in the soil and is not always related to the absorption by plants, which may depend more on plant processes. Nevertheless, it is still necessary to be vigilant about the activation effect of organic fertilizer application on As in soil.

## 5. Conclusions

Substituting chemical fertilizer with organic fertilizer will not only significantly promote the growth of water spinach but also ameliorate the ecotoxicological risks of composite heavy metals. The application of organic fertilizer significantly increased the soil pH, CEC, and OM, and decreased the available Cd and Pb, while it increased the available As in soil. Moreover, the organic fertilizer had a greater potential to reduce the Cd in vegetables compared to As and Pb. The organic fertilizer can reduce the Cd and Pb content in water spinach to below the safety limits when the added Cd in soil was below 2 mg/kg and Pb was below or equal to 90 mg/kg. Therefore, the remediation of agricultural soil by organic fertilizers should accurately consider the types and contents of pollutants in the soil, and it should evaluate the remediation effect of plants and soil as a system.

## Figures and Tables

**Table 1:** Soil-added HMs concentrations and contamination levels.

Treatment	Cd (mg/kg)	As (mg/kg)	Pb (mg/kg)	Contamination Level
S1	0	0	0	-
S2	0.1	20	50	Below RSV
S3	0.3	40	90	Equal to RSV
S4	1.0	100	300	Between RSV and RIV
S5	2.0	150	500	Equal to RIV
S6	5.0	200	1000	Higher than RIV

**Table 2:** Fertilizer application amounts of different treatments (g/pot).

Treatment	Urea	Potassium Chloride	Superphosphate	Organic Fertilizer
OF0	1.03	0.27	0.99	0
OF50	0.52	0.14	0.50	60
OF100	0	0	0	120

**Table 3:** Biomass of edible parts of water spinach (g/plant FW).

Treatments	OF0	OF50	OF100
S1	12.05 ± 0.66 aB	14.82 ± 1.15 aB	14.88 ± 1.06 aB
S2	14.81 ± 1.78 aAB	14.70 ± 0.84 aB	16.96 ± 0.28 aAB
S3	16.06 ± 0.62 bA	17.29 ± 0.40 abAB	18.36 ± 0.10 aAB
S4	13.72 ± 0.52 bAB	17.84 ± 0.28 abA	19.73 ± 1.62 aA
S5	5.83 ± 0.37 cC	11.12 ± 0.68 bC	14.88 ± 0.92 aB
S6	1.11 ± 0.10 aD	2.62 ± 0.59 aD	1.75 ± 0.11 aC

Different lowercase and uppercase letters represent significant differences for different fertilizer treatments and HMs treatments, respectively (*p* < 0.05).

## Data Availability

The data presented in this study are available on request from the corresponding author.
